# Effects of hydrolyzed yeast on growth performance, intestinal redox homeostasis, and woody breast myopathy in heat-stressed broilers

**DOI:** 10.3389/fvets.2024.1484150

**Published:** 2024-11-18

**Authors:** Abdulaziz A. Al-Abdullatif, Rashed A. Alhotan, Mohammed A. Al-Badwi, Xinyang Dong, Hannele Kettunen, Juhani Vuorenmaa, Shimaa A. Sakr, Mahmoud M. Azzam

**Affiliations:** ^1^Animal Production Department, College of Food and Agriculture Sciences, King Saud University, Riyadh, Saudi Arabia; ^2^Animal Science College, Zhejiang University, Zijingang Campus, Hangzhou, China; ^3^Hankkija Oy, Hyvinkää, Finland; ^4^Department of Animal Wealth Development, Faculty of Veterinary Medicine, Mansoura University, Mansoura, Egypt

**Keywords:** broilers, hydrolyzed yeast, intestinal redox and health, woody breast, heat stress

## Abstract

The objective of this study was to enhance the knowledge about the effects of hydrolyzed yeast supplementation on growth performance, woody breast myopathy, and its mechanism on intestinal homeostasis using antioxidant and immunomodulatory-related gene expressions in heat-stressed broiler chickens. In a 35-d feeding experiment, 160-day-old male Ross 308 broiler chickens were assigned to four dietary groups, consisting of eight replicates and five birds per replicate. Experimental diets contained four levels of hydrolyzed yeast (HY) (0, 400, 800, or 1,200 mg.kg^−1^) derived from *Saccharomyces cerevisiae*. On d 25, birds were exposed to cyclic heat stress (HS) (35°C for 8 h/d from 8 a.m. to 4.00 p.m.) for 10 days. Adding HY at 800 mg.kg^−1^ numerically decreased the feed conversion ratio (FCR) on days 25–35 (heat stress period) by 2.50%. Furthermore, the addition of HY reduced (*P* = 0.005) mortality rate compared with those of birds fed the control diet. Supplementation of HY exhibited efficacy (*P* = 0.09) in diminishing woody breast (WB) in terms of incidence and degree of severity. Furthermore, the added HY decreased (*P* < 0.001) drip loss values of the *Pectoralis major* compared with the control diet group; the addition of HY at 400 and 1,200 mg.kg^−1^ decreased (*P* < 0.001) cooking loss values in the *Pectoralis major*. In addition, HY supplementation at 800 mg.kg^−1^ decreased (*P* = 0.04) the duodenal mRNA expression of the avian β-defensin 10 (AvBD10) and increased (*P* < 0.05) the mRNA expression of nuclear factor erythroid 2–related factor 2 (Nrf2), nuclear factor kappa-light-chain-enhancer of activated B cells (NF-κB), and secreted immunoglobulin A (sIgA). The addition of HY at 400 and 800 mg.kg^−1^ decreased (*P* = 0.001) the duodenal mRNA expression of copper and zinc superoxide dismutase (Cu-ZnSOD1). HY supplementation tended to decrease (*P* =0.07) the duodenal mRNA expression of heat shock protein 70 (HSP70). The results suggest that hydrolyzed yeast supplementation to broiler chickens exposed to heat stress might improve intestinal redox homeostasis and decrease the mortality rate. The inclusion of 800 mg.kg^−1^ HY in the diet enhanced duodenal redox homeostasis, while 400–1,200 mg.kg^−1^ HY reduced mortality rate and exhibited lower drip loss values and reduced woody breast of *Pectoralis major* in terms of incidence and degree of severity.

## 1 Introduction

Postbiotics are a promising alternative to antimicrobial growth promoters (AGPs) owing to increasing concerns regarding the spread of antimicrobial resistance, and they have physiological benefits to the host either directly or indirectly (1–5). The term postbiotics has been employed to define inanimate microorganisms or soluble factors (metabolic products or byproducts) secreted by live microorganisms or released after their lysis (4). Hydrolyzed yeast, which originates from *Saccharomyces cerevisiae*, has attracted much attention as a feed supplement that consists of the total content of the yeast residue from the lysis process. Thus, it contains nucleotides, ß-glucan, B vitamins, mannan oligosaccharides, and amino acids (6, 7). Hydrolyzed yeast has a cost-effective advantage over other yeast byproducts and its feature to support beneficial interactions within the digestive and immune systems of animal due to its low molecular weight contributing to higher solubility in aqueous media makes it a promising feed additive (7, 8). Recently, it has been found that hydrolyzed yeast supplementation improved performance, meat quality, and antioxidant status and decreased *Escherichia coli* in broiler chickens (8–11).

There is a growing interest in expanding studies to mitigate the adverse effects of thermal stress on fast-growing broiler chickens as the global annual environmental temperature continues to rise. Heat stress affects meat quality, particularly through increased oxidation of proteins that reduced protein accumulation and increased fat accumulation in breast meat (12, 13), which makes proteins in the breast filets susceptible to oxidative stress and lowers their ability to bind water, resulting in increased cooking losses (10). Heat stress negatively affects cooking loss (CL) and drip loss (DL) in meat of broilers (12–14). In addition, the relationships between woody breast muscle myopathy and intestinal homeostasis have been reported (15–17). Recently, it has been found that heat stress independent of feed depression can induce significant differences in the duodenal metabolome of broiler chickens (18). It is well-acknowledged that heat stress reduces the immunological robustness, in terms of decreased secretory IgA (sIgA) and increased loads of intestinal *Escherichia coli, Salmonella, Clostridium perfringens*, and coliforms, and decreased numbers of *Lactobacillus* and *Bifidobacterium*; and such imbalance leads to the higher mortality rate (12, 19). Furthermore, heat stress affects the avian beta-defensins (AvBDs) (20) and induces oxidative stress resulting in cellular damage and inflammatory reactions, ultimately compromising growth performance and increasing energy expenditure due to the need of bird for oxidative stress mitigation and thermoregulation (20–24).

Our hypothesis was that the supplementation of dietary hydrolyzed yeast could promote intestinal health in heat-stressed broiler chickens. To our knowledge, no other reports exist regarding the impacts of dietary hydrolyzed yeast on growth performance, intestinal redox homeostasis, and woody breast myopathy in heat-stressed broiler chickens. The objective of the present study was to enhance the knowledge about the effects of hydrolyzed yeast supplementation on woody breast myopathy and its mechanism on intestinal homeostasis using antioxidant and immunomodulatory-related gene expressions in heat-stressed broiler chickens.

## 2 Materials and methods

### 2.1 Birds, housing, and experimental diets

In total, 160 1–day–old male Ross 308 broiler chickens were obtained from a commercial hatchery after they were vaccinated for infectious bronchitis and infectious bursal disease and were housed in an environmentally controlled room at the research unit of the Animal Production Department, King Saud University, Riyadh, Saudi Arabia, in wire battery cages, and each cage (58 × 50 × 35) was furnished with a radiant heater, a linear feeder, and a nipple drinker. Mash diets and water were administered *ad libitum* for the duration of the study (35 d). Mortality was recorded daily, and the percentage mortality rate was calculated for the starter phase (0–10 d), grower phase (11–24 d), and finisher phase (25–35 d). Broilers were assigned to four dietary groups in a randomized complete block design, consisting of eight replicates (cages) and five birds per replicate. Experimental diets contained four levels of yeast hydrolysate supplementation (0, 400, 800, or 1,200 mg.kg^−1^) derived from *Saccharomyces cerevisiae* (PROGUT^®^ EXTRA; Hankkija Oy, Hyvinkää, Finland). Progut^®^ extra was synthesized from spent brewery yeast through a rigorous process of strong acid hydrolysis, resulting in a final produced product that exhibits 70% solubility in water. No fraction of the hydrolysate is extricated after the hydrolysis, thereby ensuring that the product encompasses all bioactive components present within the yeast cells, including manno-oligosaccharides, β-glucan, peptides, and chitin structures. There were three-feeding phases: starter (0–10 d), grower (11–24 d), and finisher (25–35 d), and all diets were corn–soybean based (Table 1), and diets were analyzed using AOAC methods for proximate analysis (25) and HPLC for amino acids according to (26, 27). All diets were free of antimicrobial growth promotion.

**Table 1:** Levels of ingredients and nutrients of basal control diet at different phases of growth.

**Ingredients, %**	**d 1–10**	**d 11–24**	**d 25–35**
Corn	528.60	575.80	616.50
Soybean meal, 48%	391	339.8	291.0
Plant oil	37.2	44.10	52.70
Dicalcium phosphate	18.2	16.30	14.70
Limestone	10.0	9.30	8.60
Salt	4.20	3.20	3.30
Sodium bicarbonate	0.10	1.40	3.50
L-Lysin HCL	2.0	1.90	1.90
DL-Methionine	3.50	3.20	2.90
L-Threonine	1.30	1.10	0.90
Choline Cl, 60%	0.90	0.90	1.00
Premix^a^	3.00	3.00	3.00
Total	1,000	1,000	1,000
**Content of nutrients, %**			
Crude protein	23.29 (23.20)	21.15 (21.70)	19.09 (19.5)^b^
Crude fat	6.51 (6.70)	7.26 (7.20)	8.16 (8.00)
Crude fiber	2.83 (3.30)	2.72 (3.27)	2.61 (2.80)
Metabolizable energy, kcal/kg	3,000	3,100	3,200
Calcium	0.96	0.87	0.79
Available phosphorus	0.48	0.44	0.4
Digestible lysine	1.28 (1.49)	1.15 (1.32)	1.03 (1.22)
Digestible methionine	0.66 (0.70)	0.60 (0.61)	0.55 (0.56)
Digestible threonine	0.86 (1.02)	0.77 (0.92)	0.69 (0.81)

^a^Premix Per 1 Kg: thiamine (B1), 2 mg; riboflavin (B2), 6 mg; niacin (B3), 50 mg; pantothenic acid (B5), 15 mg; pyridoxine (B6), 3 mg; biotin (B7), 150 μg; folic acid, 1.75 mg; cobalamins (B12), 16.0 μg; K3 (MNB), 3 mg; D3 (cholecalciferol), 5,000 IU; A (retinol acetate), 10,000 IU; E (Dl-alpha-tocopheryl acetate), 50 IU; total antioxidants, 50; manganese (oxide), 120; zinc (oxide), 100; iron (sulfate), 40; copper (sulfate), 16; iodine (potassium iodide), 1.25; selenium (sodium selenite), 0.30.

^b^Values in parentheses are the analyzed contents.

### 2.2 Environmental data and heat stress

The ambient temperature (AM) was decreased gradually from 33°C for d 1 to 4, 30.5°C for d 5 to 7, 28.5°C for d 8 to 10, 27°C for d 11 to 15, and 24°C for d 16 to 24. A relative humidity (RH) of 33–42% was recorded, and a temperature–humidity index (THI) was calculated. On d 25, birds were exposed to chronic cyclic heat stress (35°C for 8 h/d from 8 a.m. to 4.00 p.m.) to mimic hot arid environments in broiler houses in Saudi Arabia as our laboratory previously suggested (28). The environmental temperature and humidity were recorded using EasyLog USB data loggers (Lascar Electronics, Whiteparish, Wiltshire, UK).

### 2.3 Sample collection and measurements

Body weights and feed intake were measured on d 0, 10, 24, and 35 on a cage basis. Feed conversion ratio (FCR) was calculated considering the weight of dead birds as feed intake divided by the body weight gain (g/g). The rectal temperature (°C) and respiratory rate (breaths/min) were measured on d 24 (before heat stress) and d 35 (after 10 d of heat exposure). After 10 d of heat exposure (d 35), birds close to the average weight were slaughtered as proposed in (28), and then, the carcasses were kept at 4°C in a refrigerator for 24 h. The weights of hot and cold carcasses and meat main portions (breast, legs, and wings) were measured to determine the absolute weights and relative weights as a percentage of body weights. The left breast muscles (*Pectoralis major*) were used for measuring meat quality (pH, drip-losing rate, and cooking loss). Muscle pH at 15 min and 24 h post-mortem was measured using a probe pH meter (Model: HI 8242C, Hanna Instruments Science and Technology, Beijing, China), and each breast was measured in duplicate at different locations and the values were averaged. The probe was calibrated before measuring using buffer solutions (4.01, 7.00, and 9.21) at room temperature. The initial and ultimate surface color of breast meat was determined using a colorimeter (CR-400 Chroma Meter, Konica Minolta, Tokyo, Japan). CIELAB: L^*^ (lightness), a^*^ (redness), and b^*^ (yellowness) values were measured. After deboning, the entire breast muscles (*Pectoralis major*) were collected and the degree of hardness for woody breast (WB) was graded and scaled by hand palpation and scored using normal (0), mild (1), moderate (2), and severe (3) as proposed in Tijare et al. (29). After evaluation, filet samples were packed in bags (PA/PE, 90 μm) and stored at −20°C for further analysis. The water-holding capacity of meat has been measured by determining drip loss and cooking loss of the raw meat using two replicates of each sample. To measure cooking loss, the meat samples were defrosted at 4°C for 24 h and filets were cooked at 200 °C until the internal temperature of the meats reached 70 °C in an oven-searing (TRO45RDG-B5, Black and Decker Manufacturing Company, China). After cooking, samples were taken out, cooled at room temperature, wiped gently with paper towels, and weighed again. The values expressed as a percentage of the starting weight of sample before cooking as described by (30). To determine drip loss, the meat samples (~20 g) were weighed and immediately placed in a transparent polythene bag, hung from a hook, and stored at 4°C for 1 d. After storage, the sample was wiped gently with paper towels and weighed again. The values are expressed as a percentage as the percentage of the initial muscle weight ([(W1 – W2)/W1] × 100) (31).

### 2.4 Duodenal mRNA expression assay

A part of the middle section of duodenum tissues (~50 mg) were collected from one bird per replicate and rinsed with cold phosphate-buffered saline, were snap-frozen with liquid nitrogen, and were stored at −80°C. Total RNA was isolated from duodenal tissues using a reagent (the PureLink RNA Mini Kit) according to the manufacturer's instructions (Invitrogen, Carlsbad, CA). The procedures described by (32) were used to examine duodenal mRNA expression. The endogenous reference gene glyceraldehyde-3-phosphate dehydrogenase (GAPDH) was used, and primers based on chicken sequences were synthesized at Macrogen (Seoul, South Korea) as presented in Table 2. The PCRs were performed in duplicate, and the findings were normalized to GAPDH mRNA expression. Average gene expression relative to the endogenous control for each sample was calculated using the 2^−ΔΔCt^ method (33).

**Table 2:** Sequences of primer pairs used for amplification of target and reference genes.

**Gene name^a^**	**(5^′^-3^′^) Primer sequence (5^′^-3^′^)**	**GenBank accession no**.	**Amplicon size (bp)**
GAPDH	F: CCTCTCTGGCAAAGTCCAAG	NM_204305	200
	R: CATCTGCCCATTTGATGTTG		
Nrf2	F: GGGACGGTGACACAGGAACAAC	NM_205117.1	93
	R: GCTCTCCACAGCGGGAAATCAG		
NFK-β	F: TCAACGCAGGACCTAAAGACAT	NM205134	162
	R: GCAGATAGCCAAGTTCAGGATG		
SOD1	F: TTGTCTGATGGAGATCATGGCTTC	NM_205064	98
	R: TGCTTGCCTTCAGGATTAAAGTGAG		
GPX1	F: GATGAGATCCTGAGAGTGGTGGAC	NM_001277853	123
	R: TCATCAGGTAAGGTGGGCACAA		
HSP70	F: GGGAGAGGGTTGGGCTAGAG	J02579	55
	R: TTGCCTCCTGCCCAATCA		
sIgA	F: GTCACCGTCACCTGGACTACA	S40610	192
	R: ACCGATGGTCTCCTTCACATC		
AVBD10	F: TGGGGCACGCAGTCCACAAC	NM_001001609.2	157
	R: CATGCCCCAGCACGGCAGAA		

### 2.5 Statistical analysis

The data were analyzed with a one-way ANOVA, Tukey's range test was adopted to compare means, and the woody breast data were subjected to the Kruskal–Wallis non-parametric test. Data were expressed as the mean ± SE, and it was expressed as significant when the *p*-value was < 0.05 (*p* < 0.05). Student's *t*-test was used to examine the differences between d 24 (before heat stress) and d 35 (the 10th day of heat exposure).

## 3 Results

### 3.1 Environmental data

The ambient temperature (AM), air relative humidity (RH), and the temperature–humidity index (THI) under a high-temperature environment are presented in Supplementary Table 1. The minimum AM, RH, and THI values were, on average, 24.50 ± 0.36, 33.50 ± 2.05%, and 71.02 ± 0.70, respectively, whereas the maximum values of AM, RH, and THI were, on average, 32.89 ± 1.43°C, 42.86 ± 0.81%, and 80.51 ± 1.66, respectively.

### 3.2 Stress indicators

The data on rectal temperature and respiratory rate are presented in Table 3. Indeed, on d 35 of age (10th day of heat exposure) birds showed an increase in rectal temperature (41.04 vs. 42.45°C; *p* < 0.001) coupled with a higher respiratory rate of 59.06 vs. 180 B/M; *p* < 0.001), while the addition of HY had no significant effect on rectal temperature or respiratory rate (*p* > 0.05).

**Table 3:** Effects of hydrolyzed yeast on rectal temperature (°C) and respiratory rate (breaths/min) on d 24 and d 35 of age^a^.

**Items**	**Hydrolyzed yeast, mg.kg** ^**−1**^				**SEM**	***P*** **-value**		
	**0.0**	**400**	**800**	**1,200**		**ANOVA**	**Linear**	**Quadratic**
**Rectal temperature**, °**C**								
d 24^†^	40.99	41.03	41.05	41.11	0.09	0.58	0.17	0.84
d 35^#^	42.43	42.46	42.49	42.45	0.14	0.97	0.82	0.71
Δ_Rectaltemperature_	+ 1.43	+ 1.43	+ 1.44	+ 1.33	0.18	0.92	0.60	0.69
**Respiratory rate, B/M**								
d 24^†^	58.75	58.48	62.99	56.03	6.19	0.73	0.585	0.45
d 35^#^	177	171	186	190	16.14	0.64	0.29	0.65
Δ_Respiratoryrate_	+ 118	+ 112	+ 122	+ 133	16.12	0.59	0.26	0.46
		**d 24** ^†^		**d 35** ^#^		**Sig** ^*^		
Δ_Rectaltemperature_		41.04 ± 0.03		42.45 ± 0.04		< 0.001		
Δ_Respiratoryrate_		59.06 ± 2.13		180 ± 5.58		< 0.001		

^a^*n* = 2 birds per replicate.

^†^Before heat exposure.

^#^The 10th day of heat exposure.

^*^Student's *t*-test was used to examine the differences between the d 24 (before heat exposure) and d 35 (the 10th day of heat exposure).

### 3.3 Growth performance

The data on growth performance are presented in Table 4. The addition of HY had no effect on body weight, weight gain, and feed intake. The addition of HY at 800 mg.kg^−1^ numerically (*P* > 0.05) decreased FCR on days 11–24, 1–24, and 25–35 (heat stress period) and the overall FCR (d 0–35) by 7.80, 6.0, 2.50, and 5.0%, respectively. The addition of HY reduced the mortality rate (*P* = 0.005) during the heat stress period (d 25–35).

**Table 4:** Effects of hydrolyzed yeast on growth performance in heat-stressed broilers^a^.

**Items**	**Hydrolyzed yeast, mg.kg** ^**−1**^				**SEM**	***P*** **-value**		
	**0.0**	**400**	**800**	**1,200**		**ANOVA**	**Linear**	**Quadratic**
**1–10 d**								
Initial BW, g	46.16	45.94	45.66	45.97	0.22	0.18	0.23	0.10
BW d 10, g	245	244	235	241	8.64	0.64	0.41	0.60
FI, g/b	239	237	228	229	8.28	0.47	0.16	0.71
BWG, g	199	198	189	195	8.70	0.67	0.43	0.63
FCR, g/g	1.21	1.20	1.21	1.18	0.04	0.88	0.53	0.74
**11–24 d**								
FI, g/b	1,257	1,198	1,192	1,269	54.37	0.37	0.86	0.08
BWG, g	1,099	1,094	1,123	1,151	46.61	0.60	0.21	0.86
FCR, g/g	1.15	1.10	1.06	1.10	0.03	0.20	0.184	0.10
Improvement, %^b^			−7.80					
**1–24 d**								
BW d 24, g	1,298	1,293	1,312	1,346	49.66	0.71	0.30	0.58
FI, g/b	1,496	1,435	1,420	1,499	58.19	0.41	0.97	0.09
BWG, g	1,251	1,247	1,267	1,300	49.80	0.71	0.30	0.59
FCR, g/g	1.20	1.15	1.12	1.15	0.03	0.21	0.14	0.13
Improvement, %^b^			−6.00					
**25–35 d (Heat stress environments)**								
FI, g/b	1,414	1,365	1,440	1,456	71.92	0.61	0.38	0.53
BWG, g	880	839	931	917	68.73	0.54	0.36	0.77
FCR, g/g	1.63	1.65	1.59	1.60	0.11	0.94	0.64	0.94
Improvement, %^b^			−2.50					
**1–35 d (Total growth period)**								
BW, d 35 (g)	2,178	2,131	2,243	2,263	85.37	0.41	0.18	0.56
FI, g/b	2,910	2,799	2,860	2,955	1.14	0.57	0.59	0.21
BWG, g	2,131	2,085	2,197	2,217	85.48	0.41	0.18	0.56
FCR, g/g	1.37	1.34	1.30	1.33	0.03	0.34	0.20	0.28
Improvement, %^b^			−5.00					
**Mortality rate, %**								
1–10 d	0.0	0.0	0.0	0.0				
11–24 d	0.31	0.0	0.0	0.0	0.27	0.40	0.19	0.32
25–35 d	1.87^a^	0.31^b^	0.31^b^	0.31^b^	0.47	0.005	0.004	0.02

Means ± SEM.

^a^*n* = 8 replicates per treatment, BW, body weight; FI, feed intake; BWG, body weight gain; FCR, feed conversion ratio.

^b^Improvements in relation to the control diet= mean of dietary treatment-mean of the basal control diet/mean of the basal control diet × 100.

### 3.4 Carcass traits

As presented in Table 5, the addition of HY had no significant (*P* > 0.05) effect on carcass yield, fat pad, and meat portions (breast yield, legs yield, and wings yield). In addition, giblets yield (heart, liver, and gizzard) and the index of organs (spleen, bursa, and pancreas) were not changed (*P* > 0.05) by HY application.

**Table 5:** Effects of hydrolyzed yeast on carcass yield and meat cut portions in heat-stressed broilers^a^.

**Items**	**Hydrolyzed yeast, mg.kg** ^**−1**^				**SEM**	***P*** **-value**		
	**0.0**	**400**	**800**	**1,200**		**ANOVA**	**Linear**	**Quadratic**
**Carcass yield, %**								
Hot carcass^b^	71.12	72.21	72.40	71.62	1.25	0.73	0.67	0.30
Cold carcass	70.71	71.70	71.68	71.10	1.26	0.83	0.77	0.67
**Meat cut portions and fat pad, %**								
Breasts^c^	26.81	27.99	27.47	26.85	0.85	0.46	0.88	0.14
Legs^d^	19.42	17.92	18.45	19.30	0.81	0.22	0.95	0.04
Wings	5.31	5.35	5.27	5.22	0.35	0.98	0.77	0.85
Fat pad	0.77	0.75	0.77	0.72	0.13	0.98	0.78	0.85
**Giblets yields, %**								
Liver	1.87	1.66	1.79	1.61	0.11	0.15	0.10	0.85
Heart	0.49	0.46	0.45	0.53	0.03	0.17	0.13	0.06
Gizzard	1.27	1.28	1.28	1.28	0.08	1.00	0.94	0.99

Means ± SEM.

^a^*n* = 1 bird per replicate.

^b^Excluding head, neck, feet, abdominal fat (expressed as % from BW).

^c^Whole breast = *Pectoralis* major + *Pectoralis* minor.

^d^Total leg = thigh + drumstick.

### 3.5 Quality properties of breast filets (*Pectoralis major*) and woody breast scores

The data on meat quality are presented in Table 6. The added HY decreased (*P* < 0.001) drip loss of the *Pectoralis major* compared with the control diet group. The addition of HY at 400 and 1,200 mg.kg^−1^ decreased (*P* < 0.001) cooking loss in the *Pectoralis major*. No adverse effects (*P* > 0.05) were found in response to supplement dietary HY on initial or ultimate pH and color of breast filets among the dietary treatments.

**Table 6:** Effects of hydrolyzed yeast on quality properties of *Pectoralis Major* in heat-stressed broilers.

**Items**	**Hydrolyzed yeast, mg.kg** ^**−1**^				**SEM**	***p*** **-value**		
	**0.0**	**400**	**800**	**1,200**		**ANOVA**	**Linear**	**Quadratic**
**Physical parameters of meat** ^a^								
*pH_*i*_*	6.44	6.54	6.54	6.38	0.08	0.19	0.48	0.04
*pH_*u*_*	6.14	6.14	6.15	6.18	0.04	0.75	0.34	0.61
**Initial color**_15min_								
Li^*^	50.30	50.00	50.84	49.49	0.76	0.36	0.51	0.33
ai^*^	0.22	1.32	1.282	1.442	0.61	0.19	0.07	0.29
bi^*^	11.14	9.99	10.97	10.81	0.69	0.37	0.99	0.32
**Ultimate color**_24h_								
Lu^*^	52.78	52.61	53.91	51.96	0.98	0.27	0.71	0.21
au^*^	4.10	5.22	5.09	5.25	0.56	0.15	0.07	0.24
bu^*^	14.48	13.47	14.34	14.14	0.83	0.63	0.96	0.50
Drip loss, %	2.48^a^	1.00^b^	1.10^b^	1.00^b^	0.27	< 0.001	< 0.001	0.001
Cooking loss, %	34.23^a^	30.02^bc^	33.34^ab^	28.44^c^	1.24	< 0.001	0.001	0.69
**Wooden breast myopathy** ^b^ **, %**								
0	37.50	62.50	50.00	62.50		0.71		
1	12.50	37.50	50.00	37.50		0.45		
2	25.00	0.00	0.00	0.00		0.09		
3	25.00	0.00	0.00	0.00		0.09		

Means in a row with different letters are different at *p* < 0.05.

^a^*n* = 1 bird per replicate and 3 replicate samplings (18 samples per TRT).

pHi, initial pH at 15 min; *pH*_*u*_, ultimate pH at 24 h; L^*^, lightness; a^*^, redness; b^*^, yellowness; normal, score 0; mild, score 1; moderate, score 2; severe, score 3.

^b^Data for the distribution of myopathy scores are presented as percentages. Scores are based on a 4-point scale (normal = score 0, mild = score 1, moderate = score 2, severe = score 3).

### 3.6 Woody breast scores of *Pectoralis major* muscles

**Figure 1:**
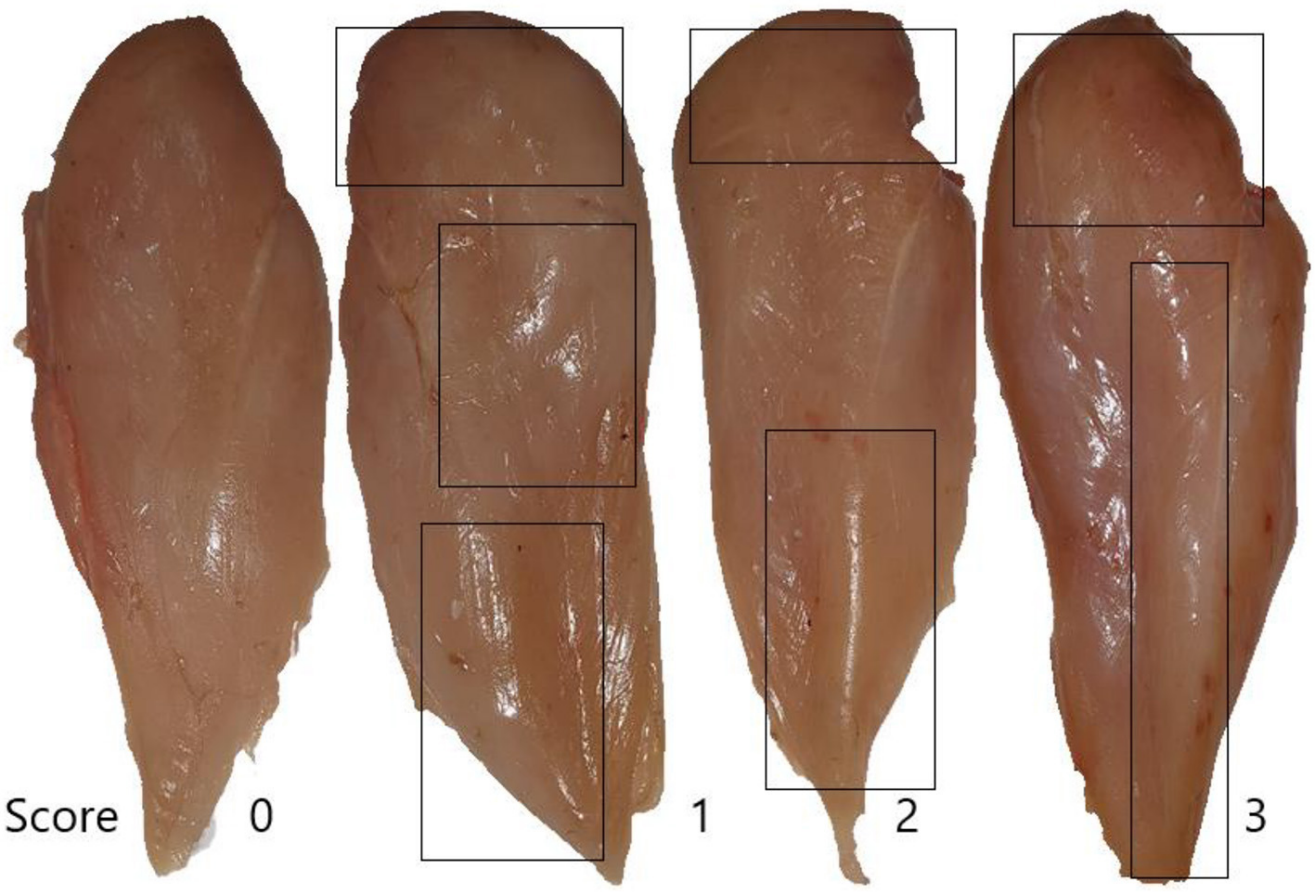
Woody scores are based on a 4-point scale (normal = score 0, mild = score 1, moderate = score 2, severe = score 3).

**Figure 2:**
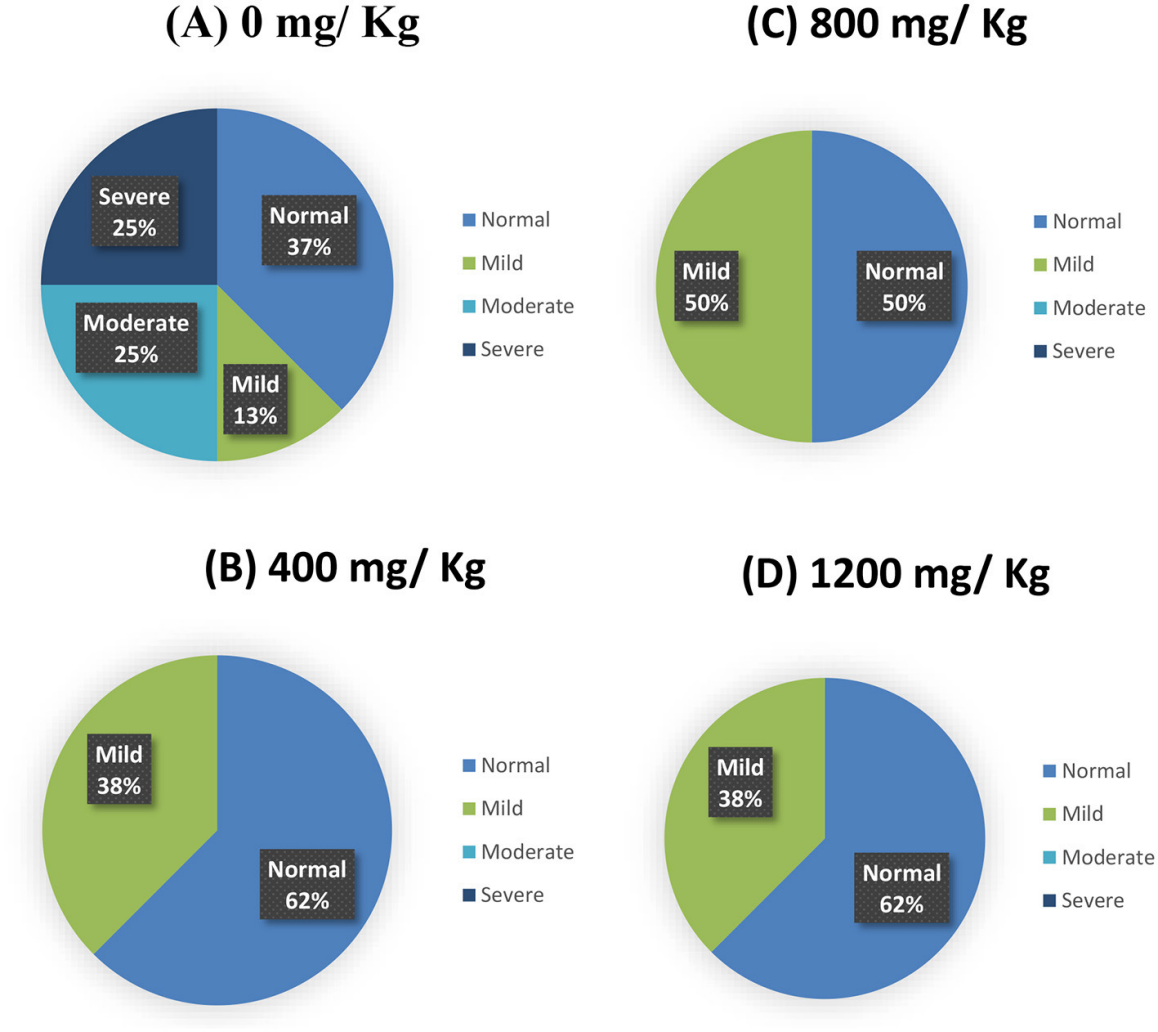
Woody breast scores (a percent distribution) for the incidence of wooden breast myopathy of male broiler chickens fed a hydrolyzed yeast. **(A)** Control diet (0); **(B)** HY at 400 mg.kg^−1^; **(C)** HY at 800 mg.kg^−1^; **(D)** HY at 1,200 mg.kg^−1^.

Woody breast myopathy scores are presented in Table 6 and Figures 1, 2. At d 35 of age, woody breast scores in all diets ranged from normal to severe. Supplementation of HY exhibited efficacy (*P* = 0.09) in diminishing woody breasts in terms of incidence and degree of severity.

### 3.7 Duodenal mRNA expression

**Figure 3:**
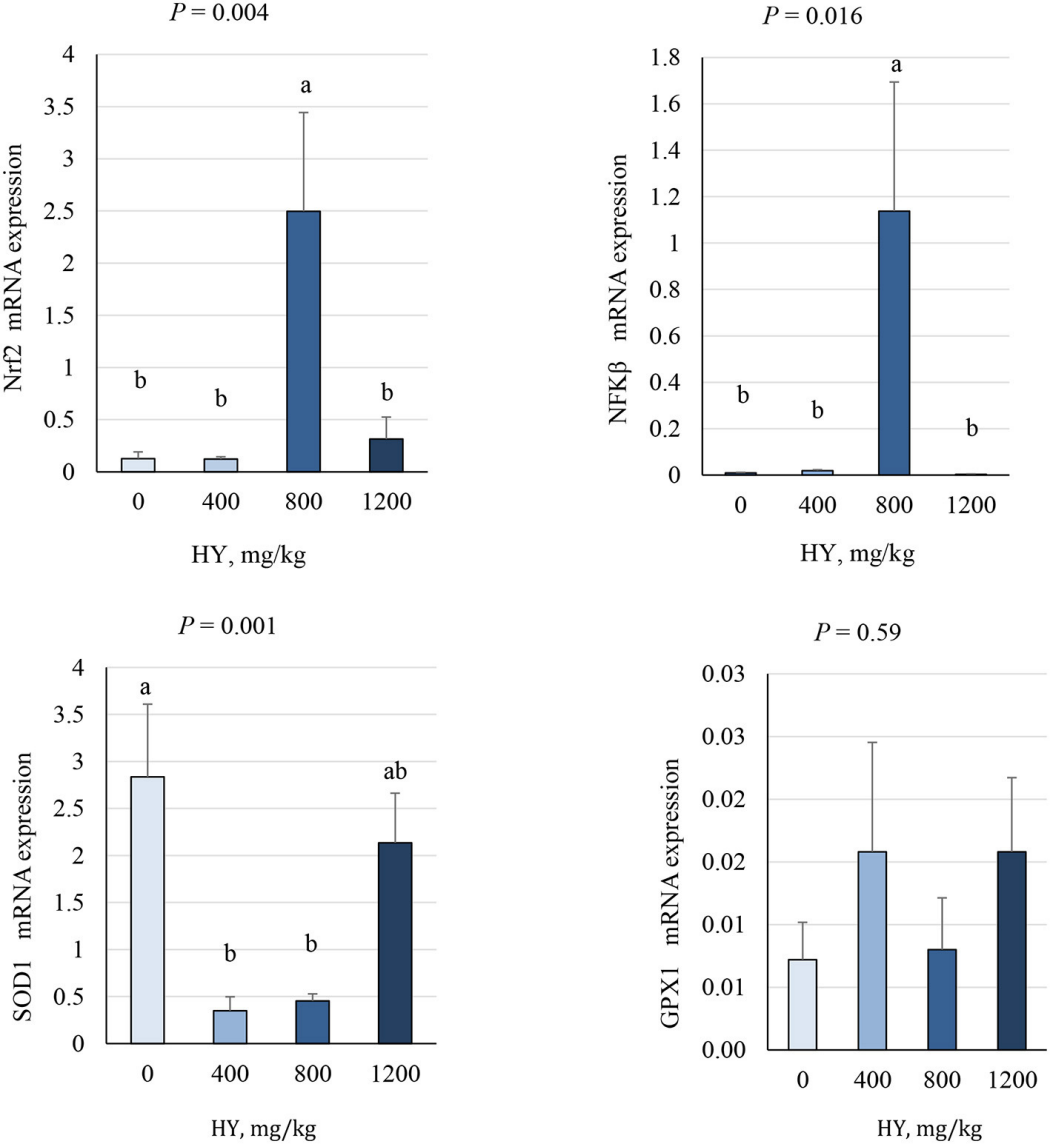
Effect of hydrolyzed yeast (HY) on the duodenal mRNA expression level of Nrf2, NF-κB, SOD1, and GPX1 of heat-stressed broiler chickens. Values are means ± SEM. Means on each bar with no common letter differ significantly at *P* < 0.05.

**Figure 4:**
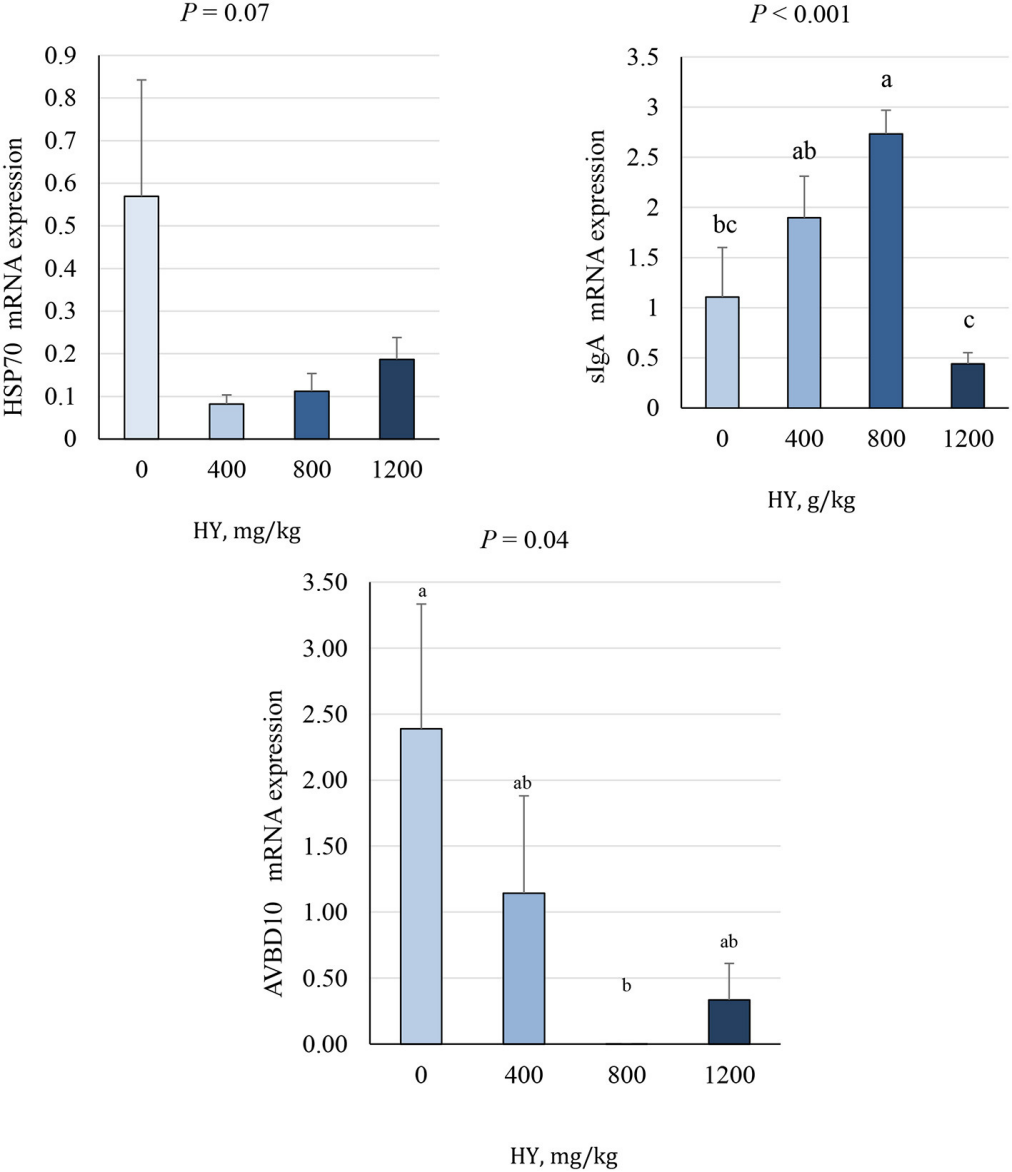
Effect of hydrolyzed yeast (HY) on the duodenal mRNA expression level of HSP70, sIgA, and AVBD10 of heat-stressed broiler chickens. Values are means ± SEM. Means on each bar with no common letter differ significantly at *P* < 0.05.

As presented in Figures 3, 4, supplementation of HY at 800 mg.kg^−1^ decreased (*P* = 0.04) duodenal mRNA expression of the avian β-defensin 10 (AvBD10) and increased (*P* < 0.05) mRNA expression of nuclear factor erythroid 2–related factor 2 (Nrf2), nuclear factor kappa-light-chain-enhancer of activated B cells (NF-κB), and secretory IgA (sIgA). The addition of HY at 400 and 800 mg.kg^−1^ decreased (*P* = 0.001) duodenal mRNA expression of copper and zinc superoxide dismutase (Cu-ZnSOD1). HY supplementation tended to decrease (*P* = 0.07) duodenal mRNA expression of HSP70.

## 4 Discussion

The temperature–humidity index and the increase, on average, in the respiratory rate and rectal temperatures confirmed that the heat stress model was able to stimulate a heat stress challenge (24, 34, 35). According to our knowledge, no study has been reported on the effect of supplemental HY on the growth performance, intestinal redox homeostasis and health, and quality properties of breast filets of broilers in environments with high temperatures. The findings presented herein indicated that the addition of HY at 800 mg.kg^−1^ decreased FCR numerically. Previous studies reported different conclusions concerning the effect of *Saccharomyces cerevisiae* hydrolysate on the growth performance of broiler chickens. A recent study reported that the addition of *Saccharomyces cerevisiae* hydrolysate at different doses (500 mg.kg^−1^, d 0–28; 250 mg.kg^−1^, d 29–42) increased broiler body weight only at 28 d (36). In addition, feeding of enzymatically treated yeast did not influence growth performance during grower and finisher phases in broiler chickens subjected to the *Eimeria* challenge (37) or did not completely alleviate the effect of the coccidia challenge on broiler chickens (38). In the present study, heat stress challenge was adopted, and this may explain why HY did not completely alleviate the negative effects of heat stress on the growth performance. It seems that the response of *Saccharomyces cerevisiae* hydrolysate differs depending on age, the amount dosage, duration included, and diseases.

In the present study, the added hydrolyzed yeast resulted in the lowest drip loss. In addition, the added hydrolyzed yeast at concentrations of 400 and 1,200 mg.kg^−1^ resulted in the lowest cooking loss (from −12 to −17%; respectively) of breast filets compared with the control diet. Recently, dietary yeast hydrolysate beneficially affected antioxidant status in blood and liver in broilers translating into lower cooking loss of breast muscle (10). Similarly, it has been found that the added *B. subtilis* fmbJ improved oxidative stability in the serum and liver, resulting in decreased cooking loss and drip loss in the breast filets of broiler chickens (39). Cooking loss and drip loss are the methods for measuring water holding capacity in the meat industry that refers to the ability of meat to keep inherent or added moisture throughout processing and cooking and, consequently, leads to better protein functionality and greater cooking yields (40, 41). It has been indicated that the gut health (microbiome) can interact with the muscle called “the intestinal microbiota–muscle axis”, leading to a possibility to improve muscle characteristics, drip-losing rate, and energy metabolism of muscle (42–45).

Heat stress affects the negative expression of essential transcription factors [e.g., nuclear factor kappa-light-chain-enhancer of activated B cells (NF-κB) and nuclear factor erythroid 2-related factor 2 (Nrf2)], both playing vital roles in regulating antioxidant and anti-inflammatory responses in poultry (46–49). An imbalance between antioxidants and pro-oxidants can mediate the activation of NF-κB and Nrf2, which triggers an antioxidant response (50, 51), ultimately increasing the energy expenditure due to the need of bird for oxidative stress mitigation and thermoregulation. Both Nrf2 and NF-κB are regulated by the redox-sensitive factors, and the activation of NF-κB has been associated with enhanced inflammatory response, while Nrf2 is involved in cellular protection against oxidative stress and inflammation (52). On the other hand, it has been reported that increased NF-κB expression during the recovery period after heat shock help the clearance of damaged proteins or regulate protein quality control after heat stress (53). Furthermore, it has been indicated that the activation of NF-κB expression during low or moderate stresses can increase the expression of Nrf2 to improve antioxidant defenses (51). Furthermore, the addition of HY at 400 and 800 mg.kg^−1^ decreased duodenal mRNA expression of SOD1. It is well-acknowledged that the activation of a range of vitagenes (e.g., SOD, GSH, and HSP70) is required to maintain optimal redox balance in the cells and a reduction in the formation of oxidants and free radical (i.e., ROS) reduces the need for antioxidant enzyme production to reduce the oxidation activity (48, 51, 54). In the present study, the increased duodenal Nrf2 and NF-κB mRNA and decreased duodenal SOD1 mRNA indicated that HY supplementation at 800 mg.kg^−1^ maintains redox (antioxidant/prooxidant) balance under heat stress in broiler chickens.

In the present study, the addition of HY decreased the mortality rate during the heat stress period. The current result is reasonable, as found herein, the ability of HY to protect cells against oxidative stress by maintaining redox (antioxidant/prooxidant) balance. It has been reported that *Saccharomyces cerevisiae* and its derivatives improved the activity of antioxidant enzymes and decreased the production of malondialdehyde (49, 55). Furthermore, a reduction in mortality rates is observed herein, considering that *Saccharomyces cerevisiae* and its derivatives have antibacterial properties and consequently can improve health status and reduce mortality of birds under heat stress (9, 56). The present results showed that supplementation of HY at 800 mg.kg^−1^ decreased duodenal mRNA expression of AvBD10 and increased mRNA expression of sIgA. Avian β-defensin 10 has antimicrobial activity against *S. typhimurium* and *E. coli* (57). The downregulation of AvBD10 indicates the absence of *S. Typhimurium* and *E. coli* infection and shows that HY might have potential antimicrobial activity against microbial pathogens and might decrease the mortality caused by pathogens. It has been reported that AvBD10 exhibited bacteriostatic activity, rather than killing microbes such as Salmonella (58). In addition, following Eimeria maxima and Clostridium perfringens, the expression of AvBD10 was detected in the jejunum of broilers (59). It has been found that oral administration of *Lactobacillus reuteri* decreased the expression of AvBD10 in the intestine (60). Recently, fenugreek seeds downregulated ileal mRNA expression of AvBD10 and altered the cecal microbial community by increasing the population of good bacteria and decreasing bad bacteria (61). Therefore, supplementation of HY might modulate the magnitude of the immune response to protect against pathogenic microbes as evidenced by the decreased duodenal mRNA expression of AvBD10 and increased mRNA expression of sIgA. Current findings are in line with previous studies observing that supplementation of HY decreased microbial *E. coli* and *Salmonella spp*. and increased *Lactobacillus spp* and sIgA (8, 9, 62). To our knowledge, this is the first study to find a relationship between hydrolyzed yeast supplementation and mRNA expression of AvBD10.

## 5 Conclusion

The results suggest that hydrolyzed yeast supplementation to broiler chickens exposed to heat stress might improve intestinal redox homeostasis and decrease the mortality rate. The inclusion of 800 mg.kg^−1^ HY in the diet enhanced duodenal redox homeostasis, whereas 400–1,200 mg.kg^−1^ HY reduced mortality rate and exhibited lower drip loss values and reduced woody breast of *Pectoralis major* in terms of incidence and degree of severity.

## Data Availability

The datasets presented in this study can be found in online repositories. The names of the repository/repositories and accession number(s) can be found in the article/Supplementary material.
