# Supplementary material for: Effects of hydrolyzed yeast on growth performance, intestinal redox homeostasis, and woody breast myopathy in heat-stressed broilers
Source: Front Vet Sci. 2024 Nov 18;11:1484150. doi: 10.3389/fvets.2024.1484150 (PMC11610250; doi:10.3389/fvets.2024.1484150)
Supplement: Supplementary file 1 [file Table_1.docx]

Supplementary data for **Figure 9A** clinical signs scores of seronegative chicks inoculated with serotype 11 (Alim IBH 1012) and serotype 8b (Alim IBH 1010) strains at different routes.

| **Experimental groups** | **Days post-infection (dpi)** | | | | | | | | | | | | | |
| --- | --- | --- | --- | --- | --- | --- | --- | --- | --- | --- | --- | --- | --- | --- |
|  | **1** | **2** | **3** | **4** | **5** | **6** | **7** | **8** | **9** | **10** | **11** | **12** | **13** | **14** |
| Serotype 11 (Oral) | 0 | 0 | 11 | 17 | 26 | 24 | 19 | 14 | 10 | 3 | 3 | 3 | 3 | 3 |
| Serotype 11 (i.m.) | 0 | 12 | 24 | 32 | 33 | 30 | 27 | 26 | 23 | 20 | 17 | 12 | 12 | 12 |
| Serotype 11 (i.p.) | 0 | 13 | 29 | 32 | 34 | 35 | 32 | 30 | 28 | 27 | 22 | 15 | 15 | 15 |
| Serotype 8b (Oral) | 0 | 0 | 13 | 18 | 20 | 19 | 15 | 8 | 0 | 0 | 0 | 0 | 0 | 0 |
| Serotype 8b (i.m.) | 0 | 9 | 17 | 20 | 22 | 25 | 21 | 19 | 17 | 11 | 7 | 3 | 3 | 3 |
| Serotype 8b (i.p.) | 0 | 11 | 19 | 22 | 26 | 28 | 25 | 22 | 17 | 15 | 12 | 6 | 6 | 6 |
| Control | 0 | 0 | 0 | 0 | 0 | 0 | 0 | 0 | 0 | 0 | 0 | 0 | 0 | 0 |

i.m.-intramuscular; i.p.-intraperitoneal

Supplementary data for **Figure 9B** day-wise mortality pattern (%) of seronegative chicks inoculated with serotype 11 (Alim IBH 1012) and serotype 8b (Alim IBH 1010) strains at different routes.

| **Experimental groups** | **Days post-infection (dpi)** | | | | | | | | | | | | | |
| --- | --- | --- | --- | --- | --- | --- | --- | --- | --- | --- | --- | --- | --- | --- |
|  | **1** | **2** | **3** | **4** | **5** | **6** | **7** | **8** | **9** | **10** | **11** | **12** | **13** | **14** |
| Serotype 11 (Oral) | 0 | 0 | 0 | 0 | 6.67 | 0 | 0 | 0 | 0 | 0 | 0 | 0 | 0 | 0 |
| Serotype 11 (i.m.) | 0 | 0 | 0 | 13.34 | 20.01 | 26.67 | 0 | 0 | 0 | 0 | 0 | 0 | 0 | 0 |
| Serotype 11 (i.p.) | 0 | 0 | 0 | 13.34 | 26.67 | 33.33 | 0 | 0 | 0 | 0 | 0 | 0 | 0 | 0 |
| Serotype 8b (Oral) | 0 | 0 | 0 | 0 | 0 | 0 | 0 | 0 | 0 | 0 | 0 | 0 | 0 | 0 |
| Serotype 8b (i.m.) | 0 | 0 | 0 | 0 | 0 | 6.67 | 0 | 0 | 0 | 0 | 0 | 0 | 0 | 0 |
| Serotype 8b (i.p.) | 0 | 0 | 0 | 0 | 6.67 | 13.33 | 0 | 0 | 0 | 0 | 0 | 0 | 0 | 0 |
| Control | 0 | 0 | 0 | 0 | 0 | 0 | 0 | 0 | 0 | 0 | 0 | 0 | 0 | 0 |

i.m.-intramuscular; i.p.-intraperitoneal
